# Practical Application of Augmented/Mixed Reality Technologies in Surgery of Abdominal Cancer Patients

**DOI:** 10.3390/jimaging8070183

**Published:** 2022-06-30

**Authors:** Vladimir M. Ivanov, Anton M. Krivtsov, Sergey V. Strelkov, Anton Yu. Smirnov, Roman Yu. Shipov, Vladimir G. Grebenkov, Valery N. Rumyantsev, Igor S. Gheleznyak, Dmitry A. Surov, Michail S. Korzhuk, Valery S. Koskin

**Affiliations:** 1 Higher School of Theoretical Mechanics and Mathematical Physics, Peter the Great Saint Petersburg Polytechnic University, 195251 St. Petersburg, Russia or anton.krivtsov@spbstu.ru (A.M.K.); sergin3d2d@gmail.com (S.V.S.); ishunpo0@gmail.com (A.Y.S.); ry.shipov@gmail.com (R.Y.S.); 2 Department & Clinic of Naval Surgery, Military Medical Academy Named after S. M. Kirov, Academic Lebedev Street 6, 194044 St. Petersburg, Russia; grebenkov_89@mail.ru (V.G.G.); doctorelanmp@bk.ru (V.N.R.); sda120675@mail.ru (D.A.S.); gensurg@mail.ru (M.S.K.); 3 Department & Clinic of Roentgenology & Radiology, Military Medical Academy Named after S. M. Kirov, Academic Lebedev Street 6, 194044 St. Petersburg, Russia; igzh@bk.ru; 4 Department of General Surgery, Omsk State Medical University, ul. Lenina, 12, 644099 Omsk, Russia; 5 Department & Clinic of Military Field Surgery, Military Medical Academy Named after S. M. Kirov, Academic Lebedev Street 6, 194044 St. Petersburg, Russia; koskinvs@gmail.com

**Keywords:** mixed reality, augmented reality, cancer, surgery, Hololens, CT scan

## Abstract

The technology of augmented and mixed reality (AR/MR) is useful in various areas of modern surgery. We considered the use of augmented and mixed reality technologies as a method of preoperative planning and intraoperative navigation in abdominal cancer patients. Practical use of AM/MR raises a range questions, which demand suitable solutions. The difficulties and obstacles we encountered in the practical use of AR/MR are presented, along with the ways we chose to overcome them. The most demonstrative case is covered in detail. The three-dimensional anatomical model obtained from the CT scan needed to be rigidly attached to the patient’s body, and therefore an invasive approach was developed, using an orthopedic pin fixed to the pelvic bones. The pin is used both similarly to an X-ray contrast marker and as a marker for augmented reality. This solution made it possible, not only to visualize the anatomical structures of the patient and the border zone of the tumor, but also to change the position of the patient during the operation. In addition, a noninvasive (skin-based) marking method was developed that allows the application of mixed and augmented reality during operation. Both techniques were used (8 clinical cases) for preoperative planning and intraoperative navigation, which allowed surgeons to verify the radicality of the operation, to have visual control of all anatomical structures near the zone of interest, and to reduce the time of surgical intervention, thereby reducing the complication rate and improving the rehabilitation period.

## 1. Introduction

Abdominal cancer patients have a high level of incidence of disease, with subsequent indications for surgical treatment. One of the most important positions is the zone of the primary location of the tumor, its volume, and direction/deepness of invasion. Moreover, the radicality of the performed surgical intervention affects the risk of recurrence and the need for reoperation [1,2]. All this determines the need for an accurate preoperative and intraoperative visualization of both the tumor itself and the anatomical structures surrounding it, taking into account their topography and the individual characteristics of the patient, especially if there was a previous surgical intervention in this anatomical zone [3,4].

As such, the issue of the development and implementation of modern methods of preoperative and intraoperative tumor and patient anatomy imaging, in order to select an effective surgical approach, remains open and requires a comprehensive discussion.

The technology of augmented and mixed reality (AR/MR) has been used in medicine for quite a long time. The current stage of medical use of AR/MR is characterized by an irreversible transition, from an educational and training tool [5,6,7,8], to the category of a surgical instrument that is used before and during surgery [9,10,11,12,13] or other medical procedures [14]. This transition is not the same in different areas of surgery [6,9,10,11,12,13,15]. In addition, researchers assessment of the role and place of AR/MR at various stages of a surgical treatment varies [14,16,17,18]. Moreover, the range of subgroups are growing within the general direction of augmented reality in surgery: transmission of optical images from video cameras of endovideosurgical or robotic devices to user’s devices of virtual reality [19], instead of traditional monitors; intraoperative transmission of images of introscopic examinations to user devices [6]; and, finally, broadcasting to augmented reality devices spatially combined with the optical image of the surgical field and the image of a three-dimensional patient model, including a dynamic one, created from introscopic data [20,21]. According to this option, the surgeon has the opportunity to have visual control of the internal anatomical structures located in the depths of the tissues. Returning to the “narrow” areas of surgery, it should be noted that publications on the application of AR/MR in cardiac surgery, urology, neurosurgery, and maxillofacial surgery are quite widely reported [10,12,16,17,22]. Publications on the use of AR/MR in abdominal surgery, especially in clinical cases with oncology, are rare [19,23,24,25].

However, the need for digital support for surgery is confirmed by the active discussion of the methods of stereotaxic surgery, including those in hybrid operating rooms, that allow performing an intraoperative CT scan [26,27,28]. Thus, the opportunities that AR/MR provides, both at the stage of preoperative planning, and especially in the intraoperative navigation are, in our opinion, extremely in demand for the surgery of primary and recurrent cancer patients; and the study of the results of applying these techniques is a part of AR/MR that is especially relevant.

The primary subject of our work is increasing the effectiveness of the surgical treatment of patients suffering from recurring malignancies of the belly and pelvis via AR/MR.

The secondary subjects are determining the problems of the perioperative application of AR/MR in early operated patients, to create an algorithm for the application AR, to determine the role of a multidisciplinary team in surgical AR/MR applications, to use invasive or non-invasive 3D model positioning, to use AR/MR during surgical procedures, to measure the deviation of a 3D model in an operative field, to estimate the results of the practical use AR/MR for difficult surgical procedures, and to share our experience for subsequent discussion.

The subsequent parts of this paper are organized as follows: Section 2 includes ethics-related statements, descriptions of research methods, clinical and technical aspects of the research material, and our algorithm of practical AR/MR. Section 3 contains a description of the action of our team regarding a surgical AR/MR application. Section 3.1 and Section 3.2 contain descriptions of clinical cases, with underlining of difficulties and obstacles we encountered in the practical use of AR/MR. Section 3.3 contains data concerning the accuracy of AR/MR in this series. Section 4 summarizes the paper as a whole and details its novel contributions. 

## 2. Materials and Methods

In this publication, we use surgical and descriptive methods. We only used medical materials, equipment, procedures, and drugs approved in the Russian Federation in the examination and treatment of all our patients. Data of clinical cases, included in description, are: diagnosis, main clinical findings, details of AR-implementing in each case, volume and duration of surgical procedures, and our impressions and other. We used no statistical test, because our patients were very different, and statistical analysis is not our goal at this time.

### Materials

The clinical part of this study’s material consists of a group of 8 patients. All patients had previous surgery. Seven were suffering from recurrent malignancies with various tumors in the abdominal and pelvic regions.

The technical part includes a hardware and software complex of augmented reality, including a personal computer running on Windows 10, Microsoft Hololens 2—mixed reality glasses, a positioning system with markers, and additional software. We used the open source 3D Slicer 4.13 software as a primary tool for DICOM data analysis and segmentation of patients’ anatomy. In addition, we developed two custom software applications. The first one was designed for the PC platform with the purpose of defining how 3D holograms were aligned with the patient using radiopaque markers and uploading these segmented 3D models to the glasses using Wi-Fi. The second program was developed for the Hololens glasses, in order to visualize this data and superimpose the 3D model using an in-built camera that tracks the marker position and orientation. We used Unity 2009.10 as a cross-platform development solution, which allowed us to design both applications for the PC and Hololens platforms. 

**Scheme 1:**
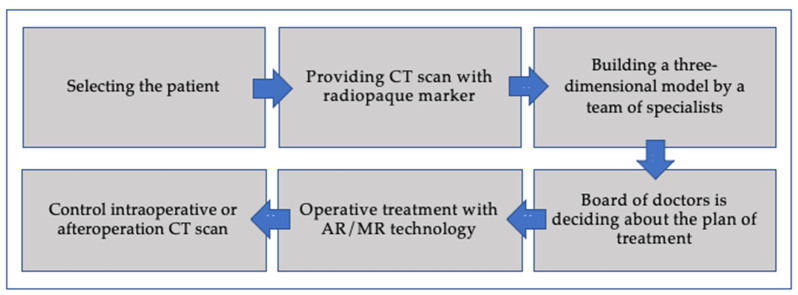
Algorithm for the application of augmented reality in surgical treatment.

The algorithm that we developed (Scheme 1) begins with the selection of the patient and providing a preoperative CT scan of the zone of interest.

The main concept is the comparison of the three-dimensional model of the patient’s anatomy with his position in the operating room. The 3D model is built by segmenting the CT data in the 3D Slicer. Augmented reality glasses are used to visualize a 3D model of the patient’s anatomy. The image in the glasses is formed by special software that allows loading the patient’s data into the glasses and linking it and comparing with a three-dimensional model using an optical label (marker) [21]. The original function of the software is an interface for the doctor, with the ability to select and disable images of anatomical structures at different stages of the operation. At the same time, control is provided by using hand gestures, which allows the user to configure the visualization of the model and maintain the sterility of the surgeon’s hands at the same time.

Two approaches were used to link the model to the patient using an optical marker. The first one is invasive using an orthopedic pin and the second is non-invasive, using adhesive magnets that act as a fixing support for the label (marker).

## 3. Results

**Scheme 2:**
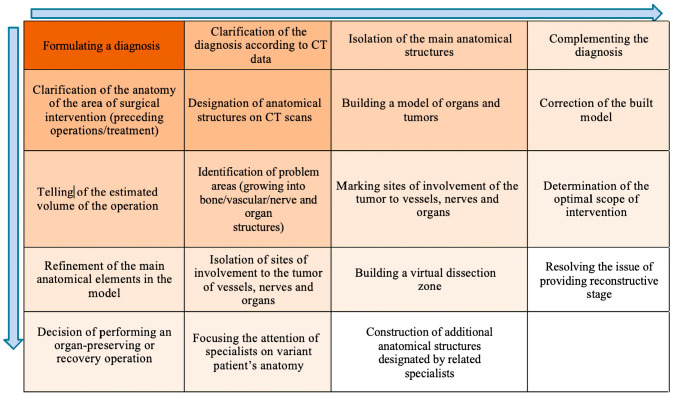
Algorithm for creating a three-dimensional model of a surgical procedure with a multidisciplinary team.

Using the method of augmented and mixed reality, we performed surgical interventions (8 clinical cases) in patients with cancer of the abdominal region (7) and with postoperative complications. Each clinical case was analyzed by a multidisciplinary team, the result was the construction and application of a situation-specific three-dimensional model. The team included a surgeon, a radiology specialist, an engineer, and related specialists (vascular surgeon, neurosurgeon, etc.). For the preoperative planning and constructing 3D model of the patients, we designed the algorithm that is presented in Scheme 2. This is one of the key steps, where segmentation of anatomical structures is performed using stage-by stage analysis of the CT data.

We present in deep detail one of our clinical cases only, as the most demonstrative. Other cases were characterized by similar clinical features, and are presented in brief form. We emphasize the development process and individual difficulties and solutions.

### 3.1. Clinical Experience in Cases with Invasive Markers

Patient T., 61 years old. Complaints at admission about constant pain in the perineal region, frequent uncontrolled urination in small quantities, feeling of an overflowing bladder. From 2018 underwent surgery, irradiation, and chemotherapy for rectal cancer.

Diagnostic results:

According to the CT of the chest, abdomen, and pelvis, a recurrence of a tumor (50 × 71 × 71 mm) was detected with signs of spread to the lower parts of the ureters, to the wall of the bladder, to the coccygeal vertebrae, as well as signs of invasion into small branches of the internal iliac arteries and the internal anterior iliac veins. There were no signs of visceral metastases.

**Figure 1:**
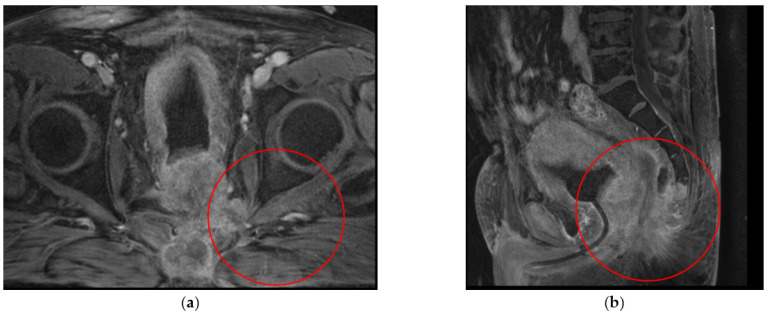
(**a**) MRI of the small pelvis with intravenous amplification—frontal projection; (**b**) MRI of the pelvis with intravenous enhancement—sagittal view (zone of tumor is circled).

According to an MRI(magnetic resonance imaging) of the small pelvis, a tumor with an approximate size of 90 × 62 × 95 mm was discovered, with signs of invasion into the prostate gland, seminal vesicles, bladder, distal ureters, presacral tissue, and coccyx, with partial destruction of the vertebrae; a bilateral hydro ureter was also found (Figure 1).

Taking into account the clinical symptoms and research data, indications were formulated for performing infralevator pelvic evisceration with distal resection of the coccyx and sacrum at the S5 level, using augmented reality technology.

Our previously proposed method [21] for attaching a marker to a frame fixed on the skull turned out to be unsuitable for this case, due to the remoteness of the anatomical zones. We designed and used a threaded pin, which was implanted in the right upper anterior iliac spine with a seat for installing an augmented reality marker. Titanium was chosen as the material for the pin. The pin was implanted on the day before operation. Next, a follow-up CT scan was performed. At the stage of performing the preoperative CT, according to which a 3D model was built, the pin was used as a radiopaque marker for the CT-study.

**Figure 2:**
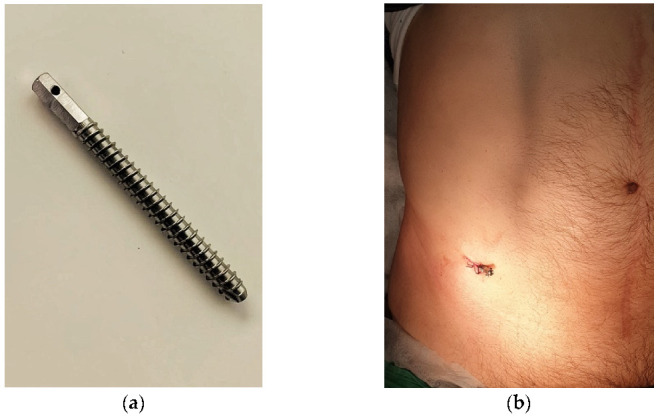

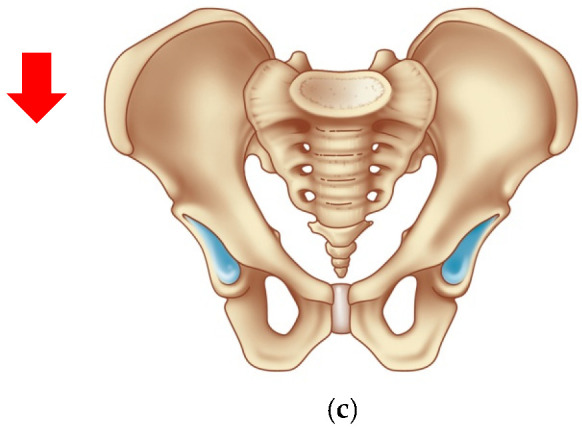
(**a**) Titanium pin with the zone for marker attachment; (**b**) View of patient with implanted pin; (**c**) Bone reference point—anterior superior iliac spine on the right.

The pin itself has a hexagonal base, with a threaded hole for fixing a removable marker, which allows one to install a sterilizable marker during the operation, as well as to use markers of various configurations, depending on the patient position on the operating table and the surgical access (Figure 2).

**Figure 3:**
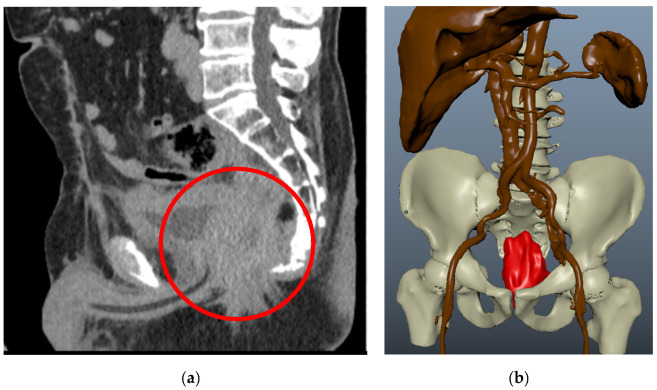
(**a**) CT scan of the pelvis with intravenous enhancement. Tumor size 50 × 71 × 71 mm, with signs of spread to the lower parts of the ureters, the wall of the bladder, and the coccygeal vertebrae (highlighted in a circle); (**b**) 3D model of pelvic organs and vessels. Implanted pin at the right side.

Based on the CT data of the abdomen and small pelvis, a virtual model of the small pelvic organs was built for additional visualization of the location and invasion of the tumor into the surrounding tissues and organs (Figure 3).

To visualize a 3D model of the patient’s anatomy in augmented reality glasses during the operation we developed special software, which allows loading the patient’s data into the glasses and to linking it to the three-dimensional model with the help of an optical marker.

The developed software uses as input data, a set of DICOM slices, which are obtained using a computed tomography scanner with preinstalled radiopaque markers. The software allows segmenting the area of interest manually and building a 3D model based on it. In addition, it is possible to load ready-made models built using different software products (e.g., 3D Slicer).

For the correct visualization of the display in the glasses, it is necessary to determine the position of the areas of interest (organs, vessels, tumor) relative to the tracking marker. The program calibrates the position and orientation of the marker using three X-ray contrast marks, which should be clearly visible. Calibration can be done automatically, according to the known configuration of the marker. Moreover, if it is impossible to use the automatic mode, a manual version is provided.

The described application running on a computer works in tandem with an application running in the glasses. After the calibration is completed, the data are prepared and transferred to the glasses for the visualization. In this case, the glasses and the computer should be connected to the same network. The application for the glasses determines the position of the marker in real space and aligns the position of the hologram according to the position obtained during the calibration.

**Figure 4:**
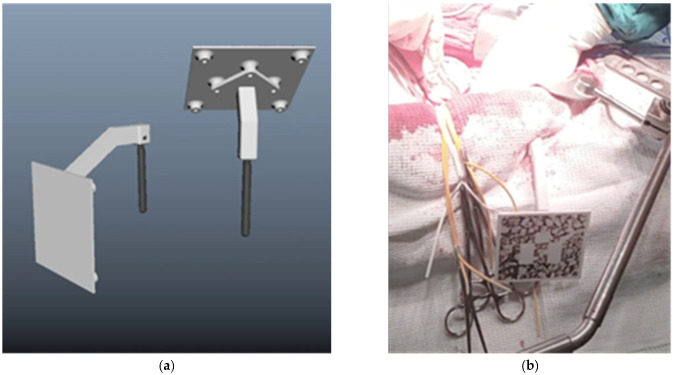
(**a**) Two configurations of the marker for augmented reality; (**b**) Sterilized and installed marker of the first (“abdominal”) configuration.

In the clinical case, taking into account the two stages of the operation: position of the patient on the back, and turning over to the prone “jack knife” position, with the help of 3D printing method two separate markers were made for each of the two positions (Figure 4). Before turning the patient, the “abdominal” marker was removed from the pin, and a “perineosacral” marker was put in its place.

**Figure 5:**
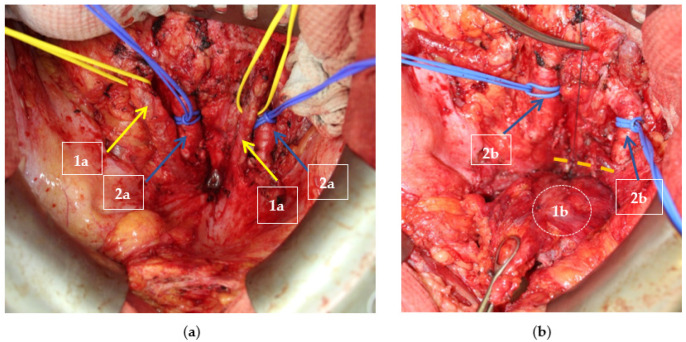
(**a**) Intraoperative view of the pelvis. Performed control of bleeding. 1a—ureters; 2a—common iliac arteries; (**b**) Resection of the ureters was performed, the bladder was mobilized, and the border of the tumor was determined using augmented reality technology. 1b—mobilized bladder; 2b—common iliac arteries. The boundary of the tumor invasion into the sacrum is indicated by a ligature and a dotted line.

The augmented reality technique made it possible to clarify the borderzone of sacral resection, determine the points of tumor fixation, perform a full washout of the pelvic cavity, and perform final hemostasis (Figure 5).

**Figure 6:**
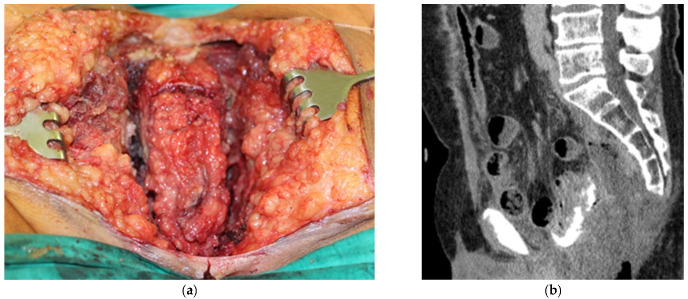
(**a**) The borders of the sacrum resection are outlined in the patient in “jackknife” position; (**b**) Postoperative CT scan.

After completing the abdominal stage of the surgical intervention, the “abdominal” marker was changed for the “perineosacral” one, and the patient was turned to the prone “jack knife” position. A stage refinement of the topographic and anatomical features of the pelvic tumor was carried out using a 3D augmented reality model, and the level of sacrum transection was corrected, followed by CT control (Figure 6).

**Figure 7:**
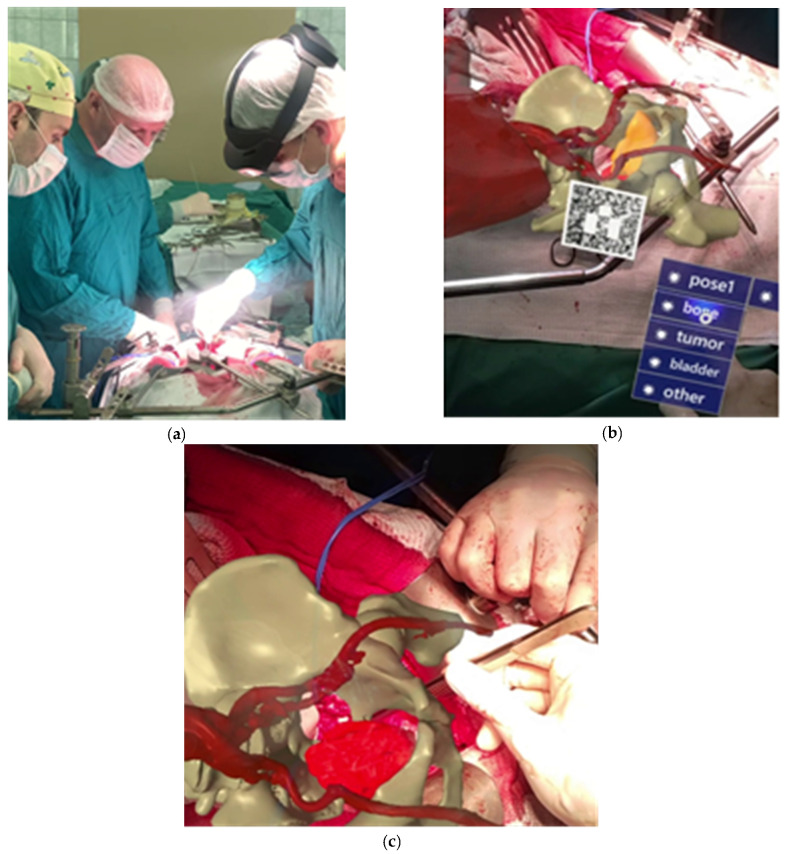
(**a**) Surgeon at work with augmented reality glasses; (**b**,**c**) Visualization of the 3D anatomy model and physician interface in the augmented reality glasses.

As a result of the stage by stage mobilization of the tumor and transection of the sacrum, the tumor was removed as a single block (Figure 7). The postoperative period proceeded without any complications.

**Figure 8:**
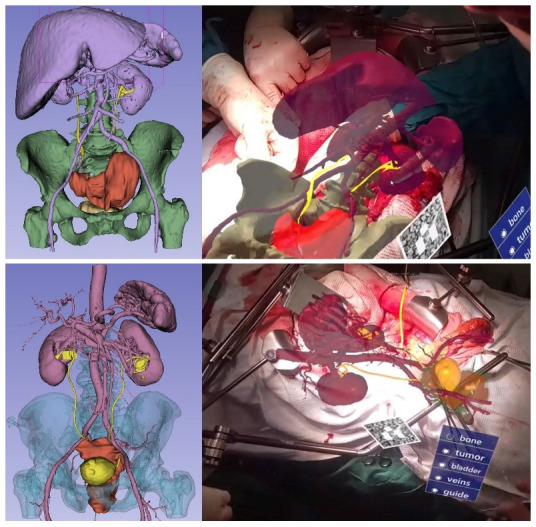
3D model of the patient anatomy reconstruction and its overlay during the operation. According to Table 1, patient 2 image is above, patient 3 image is below.

In total, three patients were operated on with the help of this technique of marker fixation (Table 1); an example of a preoperative projection of the patient’s anatomy and its intraoperative usage overlain on the surgical field is shown in Figure 8.

### 3.2. Clinical Experience in Cases with Noninvasive (Skinbased) Markers

**Figure 9:**
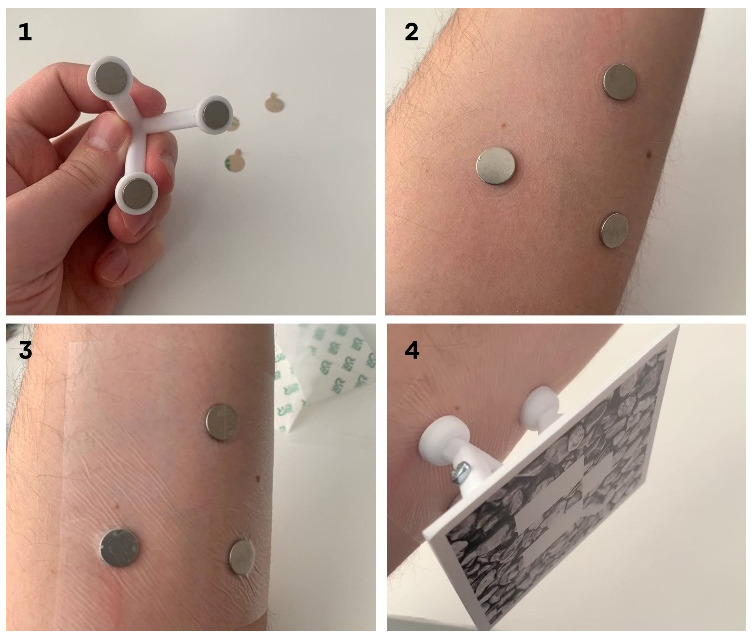
First stage—magnets are placed on the guide frame’s sockets. The protective cover for the adhesive layer on each magnet is removed. Second stage—magnets are installed on the skin using the guide frame. Third stage—magnets are covered with protective film and the patient is ready for the CT scan. Fourth stage—the sterile marker is attached on these magnets for augmented reality during surgical procedure.

Taking into account the disadvantages and features of the invasive attachment method, a noninvasive method of marking based on magnets was developed, which are attached to the skin and act as a fixation point for the marker and radiopaque marks (Figure 9).

This solution is based on the usage of neodymium magnets. Marker installation has several stages. First, the magnets are preinstalled on the guide frame, then it is pressed to the skin, together with the magnets. Due to the presence of an adhesive surface, after removing the frame, the magnets remain fixed on the skin at a predetermined position. However, the strength of the adhesive layer of the magnets is not sufficient for long-term fixation, so additional fixation with an adhesive film should be used. We used a 10 × 10 cm Suprasorb film, which covered the area of the magnets in excess and allowed them to be fixed for a long time (for up to 1 week). Then, when magnets are fixed, the patient undergoes a CT scan, where the magnets are used similarly to radiopaque markers for subsequent cooperation of the 3D model with the marker. The final step is to install a marker on these magnets for the operation. In this case, the marker is presterilized; the marker installation site with a moisture resistant film can also be sterilized.

**Figure 10:**
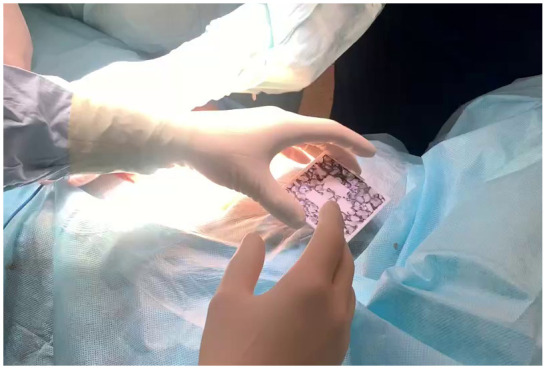
Moment of intraoperative marker placement in sterile conditions.

This approach has a number of significant advantages. First of all, it is noninvasive and it gives the ability to install a marker in any anatomical zone. Due to the sufficiently strong degree of fixation of the magnets, the installation site can be covered with sterile material and the marker can be placed on top (Figure 10).

**Figure 11:**
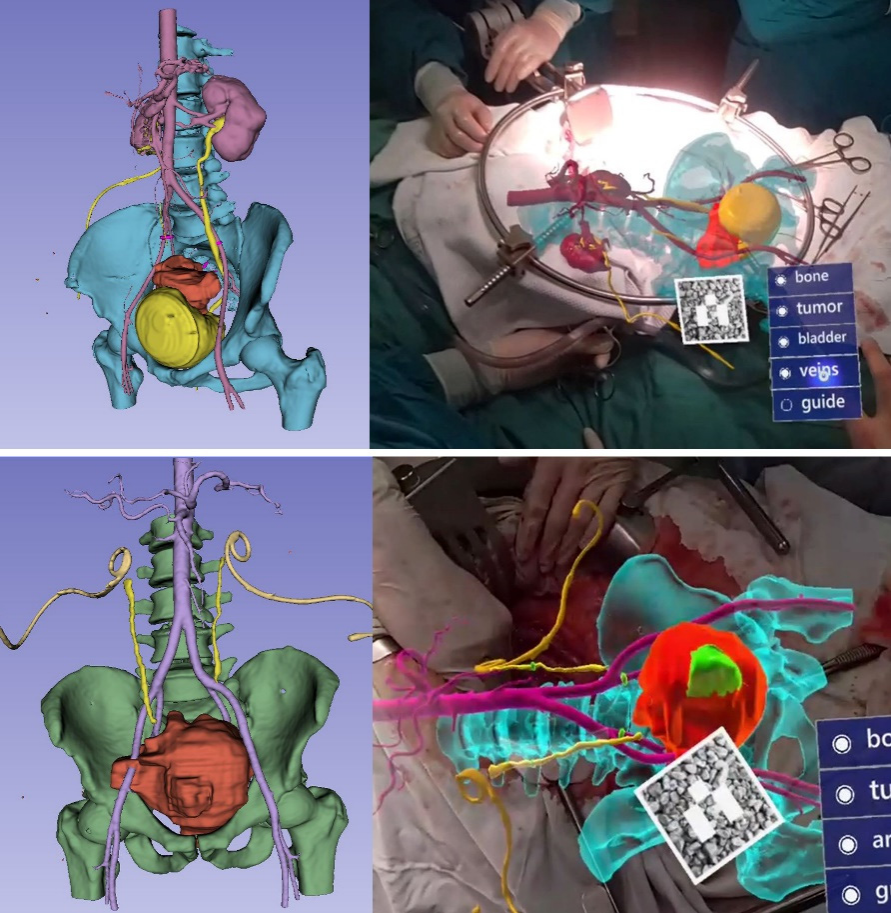
3D model of the reconstruction of the anatomy of the patient and its overlay during the operation. According to Table 2, patient 3 is above, patient 1 is below.

In addition, during the operation, the label can be removed and installed only when it becomes necessary to visualize the anatomical model in augmented reality glasses. On the other hand, the disadvantage of this solution may be a lesser accuracy, due to fixation to the movable surface of the skin. In the following patients (Table 2) who received surgical treatment, we used magnetic (on-skin) fixation of marks:

In total, five patients were operated on with magnetic (on-skin) fixation of marks (Table 2); an example of a preoperative projection of the patient’s anatomy and its intraoperative usage overlain on the surgical field is shown in Figure 11.

### 3.3. Hologram Positioning Accuracy

To assess the positioning accuracy of holograms in the augmented reality mode, a stand was designed consisting of three mutually perpendicular planes with millimeter markings applied on each of them. The size of the working area of the stand is 400 × 400 mm. The base itself was assembled on the basis of a metal frame with the ability to adjust the offset and inclination of each plane. Calibration and correction of the position of the planes was done using measuring tools: a square and ruler with a high accuracy class.

**Figure 12:**
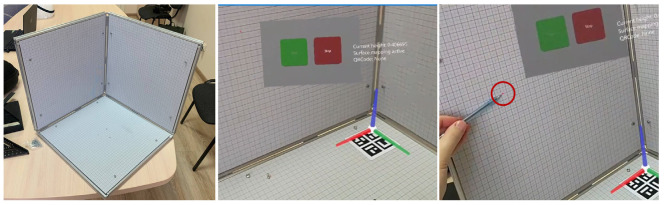
The stand for measuring positioning accuracy (left). The marker is placed in predefined spot (center). Three different 1 mm spheres are visualized on the each side of the stand. The deviation around 2–3 mm can be seen on the right image by comparing the pointer position (where the sphere should be) with actual sphere position (red dot). The step of the grid is 1 mm.

The algorithm for using the stand is based on setting a marker at the base of the stand coordinate system and visualizing 1-mm spheres on the stand planes in augmented reality. After the mixed reality glasses have recognized the marker, the observer marks the actual location of the sphere on the stand layout. The evaluation takes place in each plane of the stand in different positions of the observer relative to the marker, after which the displacement of the marked points is compared relative to the given coordinates. As a result, the average deviation within a radius of 250 mm from the marker was 2–3 mm (Figure 12).

It is also worth considering the error when comparing the marker with patients. In the first case, using an implanted pin, the error was 1–4 mm. Thus, the average accuracy level was 3–5 mm.

In the case of using a marker that is attached to magnets, the value of the matching error was approximately the same; however, in the case of skin displacement as a result of the use of wound dilators, the total error could reach up to 5 mm. If the marker was placed on the chest, the overall accuracy was up to 10 mm, the main reason being the displacement of the chest during breathing.

## 4. Discussion

The surgical interventions performed in the treatment of recurrent cancer of the abdomen and pelvis, and after other previous surgeries, are technically quite complex from a surgical perspective. They are associated with a high risk of unwanted damage of the anatomical structures, frequent massive intraoperative hemorrhage and blood-loss. They require, in addition to the high skill of the operating surgeon, thorough comprehensive preoperative planning and intraoperative visual support for verification of topographic and anatomical relationships in the area of the surgical intervention. Moreover, a lot of other questions can emerge depending on the personalized situation.

Difficulties that are associated with the individual characteristics of the patient can be successfully overcome with the help of forecasting and taking appropriate measures. Obviously, the labor costs of highly qualified personnel in the preparation and provision of an operation with this method are very significant. However, it seems to us that they are absolutely justified, since the main goal is achieved: increasing the radicality, while reducing the surgical trauma, which directly increases the effectiveness of the treatment.

In comparison to other solutions [10,12,16,17,22], the developed methods with the help of invasive and non-invasive markers provide flexibility for positioning 3D models of the anatomy on almost any body part. However, this approach has some limitations regarding the overall accuracy of hologram positioning, specifically using non-invasive magnetic markers. On top of that, the current solution does not allow the tracking of surgical tools, as the inbuilt camera struggles to track multiple markers at once and cannot maintain the same performance. These issues limit the technology mostly to use in preoperative and intraoperative planning, and do not allow it to be used as a navigation tool.

A range of recent publications concentrated attention on selected features of AR/MR, such as geometric accuracy [29,30]. In our opinion, geometric accuracy is a very important feature of any diagnostic and curative method, but not the only one. Noises and occlusions, and other obstacles, can reduce the surgical benefit of AR/MR [31]. We try to share our experience of detecting and overcoming difficulties of the practical use of AR/MR in surgery.

A short list of the proposed innovation:

- Algorithm for the application of augmented reality in surgery
- Algorithm for creating a three-dimensional model of a surgical procedure by a multidisciplinary team.
- Bone fixation of a marker
- Two (or more) marker design for position changes
- Magnet fixation of a marker
- Gestures control menu

## 5. Conclusions

The presented clinical cases and the algorithm for using augmented and mixed reality technology confirmed the fact that the use of this approach improves the accuracy of preoperative planning, and helps in determining the level of tumor spread and invasion into the surrounding anatomical structures, which increases the radicality of surgical operation and the safety of the performed surgical procedure.

In future studies, in order to increase the total positioning accuracy, we are planning to use an external optical tracking system with multiple infrared cameras. This would help us to track multiple markers at high frequency and to use a pointer or track specific surgical tool during the procedure. In addition, we are continuing to develop a non-invasive approach for marker attachment and to combine it with a new optical tracking system, where a patient’s registration and calibration will happen in the operating room using anatomical landmarks. Recalibration in the middle of the procedure can help to alleviate any errors after using wound dilators and other factors.

## Figures and Tables

**Table 1:** Patients with invasive (pin) fixation of marks.

ID	Diagnosis	Operation	Duration, min
1	Cancer of the lower rectum cT3N1M0/pT3N1aM0 Lvi(+) IIIB st. ^1^	Infralevator evisceration of the small pelvis, resection of the small intestine, cholecystectomy, resection of the coccyx, bilateral ureterocutaneostomy.	380
2	Cancer of the lower ampulla of the rectum pT3cN1aM0 IIIB st. ^1^	Infralevator evisceration of the small pelvis with distal resection of the coccyx and sacrum at the S_5_ level (removal of the tumor by a single block with the bladder, prostate gland and the distal part of the sacrum).	335
3	Ovarian cancer T3cNxM1 IVa st.^1^	Combined cytoreductive (initially optimal) operation: posterior supralevator evisceration of the small pelvis. Resection of the right dome of the diaphragm. Resection of the greater and lesser omentum. Obstructive resection of the sigmoid colon. Total peritonectomy. Cholecystectomy, splenectomy, appendectomy. Resection of the right ureter (Figure 8).	390

^1^ All patients were suffering from recurrence malignancies and had had surgery before. TNM Classification of Malignant Tumor (TNM-stage) is matched to previous surgery.

**Table 2:** Patients with noninvasive (on-skin) fixation of marks.

ID	Diagnosis	Operation	Duration, min
1	Bladder cancer T4bN3M1 (Lim) IV ^1^	Radical cystectomy. Lymph node dissection along the aorta, in the aorto-oval space, and along the course of the iliac vessels.	330
2	Cervical cancer pT1b2N0M0 Ib st. Recurrence with the formation of a vesico-vaginal and recto-vaginal fistula ^1^	Pelvic evisceration (radical cystectomy with formation of Bricker ileum conduit, resection of the vaginal stump, anterior resection of the rectum).	90
3	Cervical cancer cT2bN0M0 IIb st ^1^	Diagnostic laparotomy (revision of the abdominal cavity, revealed multiple areas of carcinomatosis throughout the abdominal cavity, performing a radical operation was technically impossible).	110
4	Hepaticocholedochal stricture	Laparotomy. The rehepaticojejunostomy with a long Roux loop.	270
5	Recurrent chondrosarcoma of the 11th rib on the left with spread to the dome of the diaphragm, left kidney ^1^	Not completed due to clinical exacerbation.	N/A

^1^ All patients were suffering from recurrent malignancies and had had surgery before. TNM-stage is matched to previous surgery.

## Data Availability

The data reported in this study are available upon requested from the corresponding author.
